# Assessment of Platelet Reactivity and Inflammatory Markers in Coronary Artery Bypass Graft Patients Treated with Acetylsalicylic Acid with Flavonoid Supplementation

**DOI:** 10.3390/molecules26247486

**Published:** 2021-12-10

**Authors:** Aldona Siennicka, Magdalena Kłysz, Monika Adamska, Kornel Chełstowski, Andrzej Biskupski, Maria Jastrzębska

**Affiliations:** 1 Department of Laboratory Diagnostics, Pomeranian Medical University, Powstańców Wlkp. 72, 70-111 Szczecin, Poland; magdalena.klysz@pum.edu.pl (M.K.); adamska.monika31@gmail.com (M.A.); kornelch@pum.edu.pl (K.C.); mariajas@pum.edu.pl (M.J.); 2 Department of Cardiac Surgery, Pomeranian Medical University, Powstańców Wlkp. 72, 70-111 Szczecin, Poland; a.biskupski@vp.pl

**Keywords:** acute-phase markers, aspirin, cardiac surgery, diosmin, platelet aggregation

## Abstract

The recommended pharmacological therapy for patients with coronary artery disease (CAD) treated by coronary artery bypass grafting (CABG) is acetylsalicylic acid (ASA). To improve the antiplatelet effect, supplementation with flavonoids is also recommended. The aim of this study was to estimate anti-aggregation properties of diosmin, in combination with ASA, pre- and postoperatively and assess the relationship of this therapy with inflammatory processes in CAD patients undergoing CABG. The study patients (*n* = 26) took diosmin (1000 mg/day); the control patients (*n* = 27) took a placebo. The therapeutic period for taking diosmin was from at least 30 days before to 30 days after CABG. All patients also took 75 mg/day ASA. Platelet aggregation and IL-6, CRP, and fibrinogen concentrations were determined before and 30 days after surgery. Results showed that diosmin did not enhance the anti-aggregation effect of ASA at any assessment time. However, there was a stronger anti-aggregation effect 30 days after surgery that was diosmin independent and was associated with acute-phase markers in the postoperative period. Increased levels of inflammatory markers in the late phase of the postoperative period may provide an unfavorable prognostic factor in long-term follow-up, which should prompt the use of stronger antiplatelet therapy in patients after CABG.

## 1. Introduction

Recommended standards of treatment for coronary artery disease (CAD) include both pharmacological and cardiosurgical treatments. Pharmacological treatment includes antiplatelet drugs, mainly with acetylsalicylic acid (ASA, aspirin), used alone or in combination with clopidogrel. However, importantly, some patients have a low response to ASA therapy, resulting in repeated thrombotic events despite treatment [1,2,3,4,5]. Due to the high clinical significance of reduced platelet sensitivity to ASA, the causes and mechanisms of reduced effectiveness of aspirin therapy are still being sought. This problem is significant in patients with coronary artery disease, especially those treated with coronary artery bypass grafting (CABG). In CAD patients who receive ASA and undergo CABG surgery, a frequent surgical complication is paroxysmal atrial fibrillation, involving up to 30% of operated patients [6,7]. Some research has indicated a relationship between atrial fibrillation and increased platelet activation, possibly related to a low response to antiplatelet therapy [8,9,10]. For this and other reasons, research assessing possible improvements to the effectiveness of aspirin therapy for CABG patients still deserves the attention of cardiac surgeons. At present, the most commonly used improvement with patients with atrial fibrillation is the addition of clopidogrel to ASA therapy [11]. To improve the antiplatelet effect of ASA therapy in patients with cardiovascular diseases, supplementation with natural or synthetic flavonoids is also recommended. Considerable interest among cardiologists and cardiac surgeons has resulted mainly from the fact that flavonoids may inhibit platelet activation and reduce the formation of platelet aggregates and are highly likely to mimic the anti-aggregating effect of aspirin, i.e., the action that inhibits arachidonic acid metabolism [12,13,14,15,16,17]. Among the flavonoids used in cardiac surgery, the most common are flavonols. It has been shown that diosmin supplementation to ASA therapy with CABG patients translates into better clinical efficacy, as assessed by the New York Heart Association (NYHA) scale in patients with left ventricular failure [18]. Although diosmin is a relatively old supplement, introduced into medicine in 1969, its characteristics are still of interest due to its diverse biological activities directed toward antiplatelet properties [19] and its antioxidant and anti-inflammatory effects [20,21]. The anti-inflammatory properties of diosmin are particularly documented in patients with venous circulatory insufficiency [19,20,22,23]. This is important in the cardiosurgical treatment of coronary artery disease because the most common source of material for CABG surgery is the saphenous vein. It could be assumed that flavonols, including diosmin, reduce the inflammatory reaction and improve the quality of the transplanted saphenous vein, thereby extending the patency of the venous bypass and thus prolonging the life of the operated patient. We are currently conducting further long-term research on the effectiveness of flavonoids on the quality of the saphenous vein used as a venous bypass in the surgical treatment of coronary artery disease. As mentioned, the adverse clinical consequence associated with CABG has been attributed to the increased platelet activation. In turn, the variety of cytokines secreted by platelets may be involved in the inflammatory response to coronary surgery. Therefore, the present study focused on the anti-aggregation properties of diosmin in combination with ASA, pre- and postoperatively, and assessed the relationship of this therapy with inflammatory processes.

Some studies have shown that increased levels of inflammatory markers, mainly interleukin-6 (IL-6) and C-reactive protein (CRP), are associated with an increased risk of perioperative complications and mortality in surgical patients [24,25,26], with limited benefit from percutaneous coronary intervention (PCI) in patients with CAD [27]. At present, the lack of a clear concept concerning the relationships between platelet activation and inflammatory processes suggests that research in this area is warranted. This is all the more interesting due to the fact that antiplatelet drugs are also endowed with anti-inflammatory properties [28,29,30].

The aims of our study were (1) to assess whether a better response occurred with ASA anti-aggregation treatment in combination with diosmin supplementation (potentially reducing aspirin resistance) in patients with coronary artery disease with CABG surgery and (2) to assess the severity of postoperative inflammation and possible relationships between acute-phase markers and the antiplatelet effects of ASA with diosmin supplementation, which could impact the risk assessment for postoperative thrombotic complications.

## 2. Results

**Figure 1:**
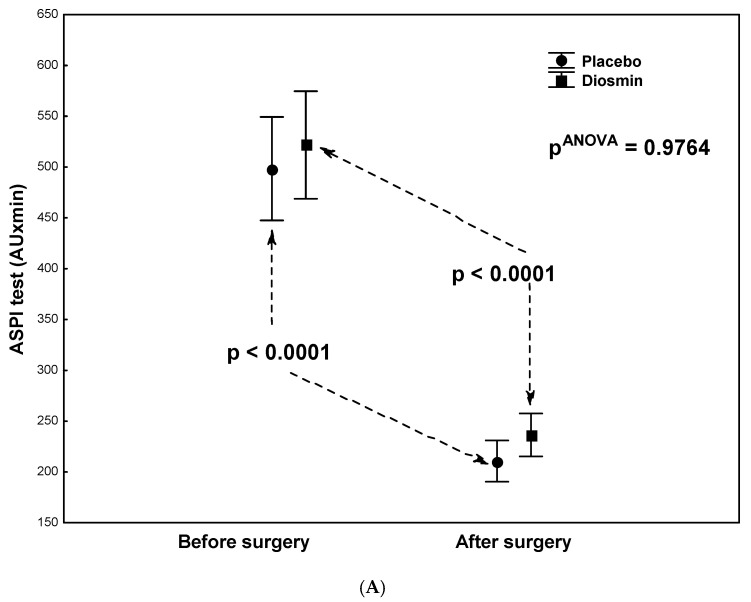

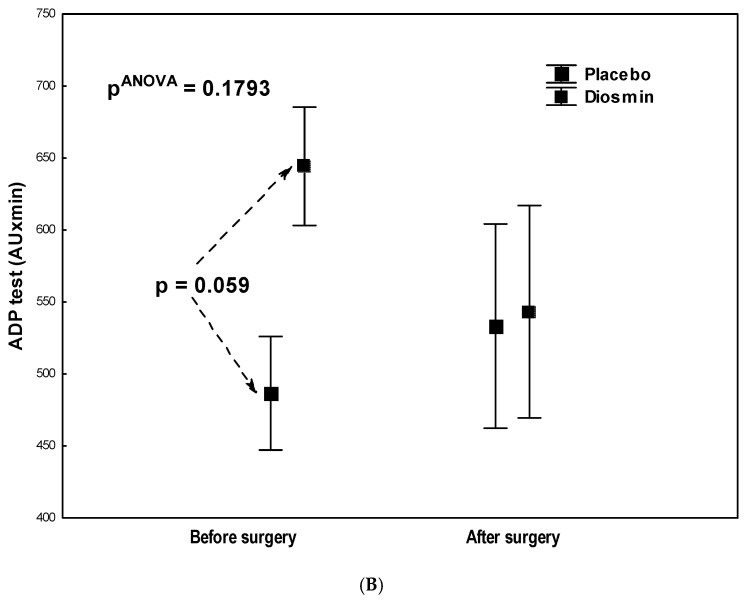
(**A**) Arachidonic-acid-induced platelet aggregation (ASPI test) results from patients who underwent acetylsalicylic acid therapy with diosmin supplementation (study group) versus patients who underwent acetylsalicylic acid therapy with a placebo (control group) at two times (before and after surgery). (**B**) ADP-induced platelet aggregation (ADP test) results from patients who underwent acetylsalicylic acid therapy with diosmin supplementation (study group) versus patients who underwent acetylsalicylic acid therapy with a placebo (control group) at two times (before and after surgery). AU, arbitrary units.

**Figure 2:**
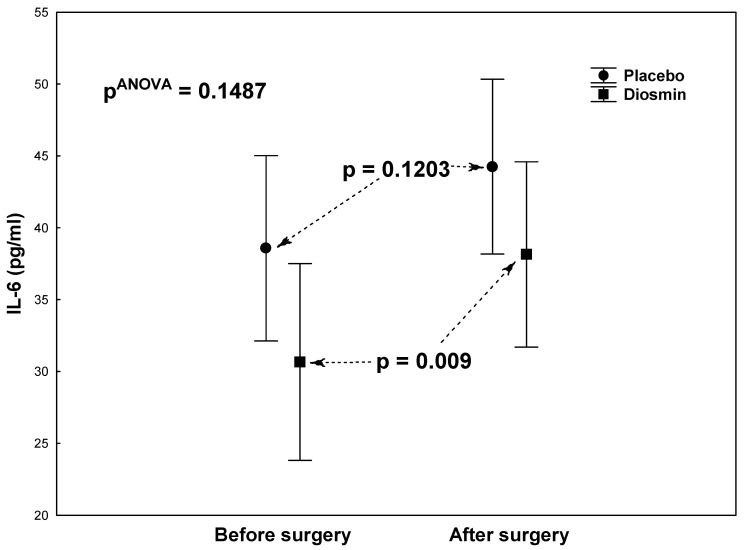
Interleukin-6 (IL-6) levels in patients who underwent acetylsalicylic acid therapy with diosmin supplementation (study group) versus patients who underwent acetylsalicylic acid therapy with a placebo (control group) at two times (before and after surgery).

**Figure 3:**
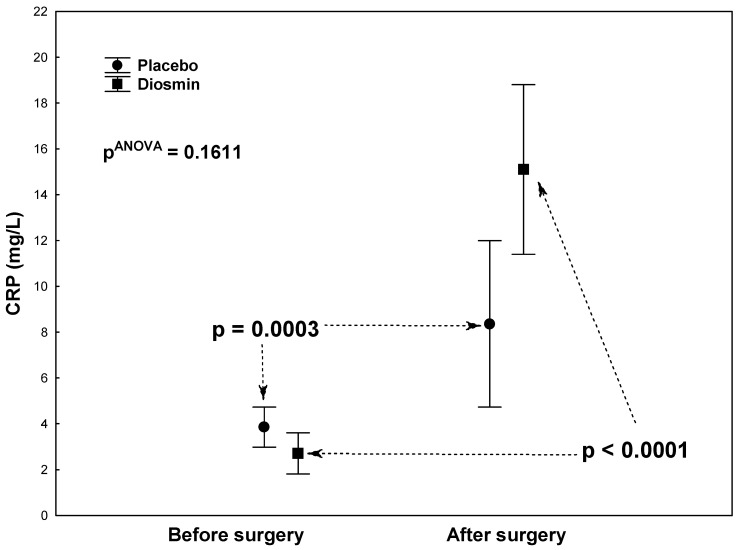
C-reactive protein (CRP) levels in patients who underwent acetylsalicylic acid therapy with diosmin supplementation (study group) versus patients who underwent acetylsalicylic acid therapy with a placebo (control group) at two times (before and after surgery).

**Figure 4:**
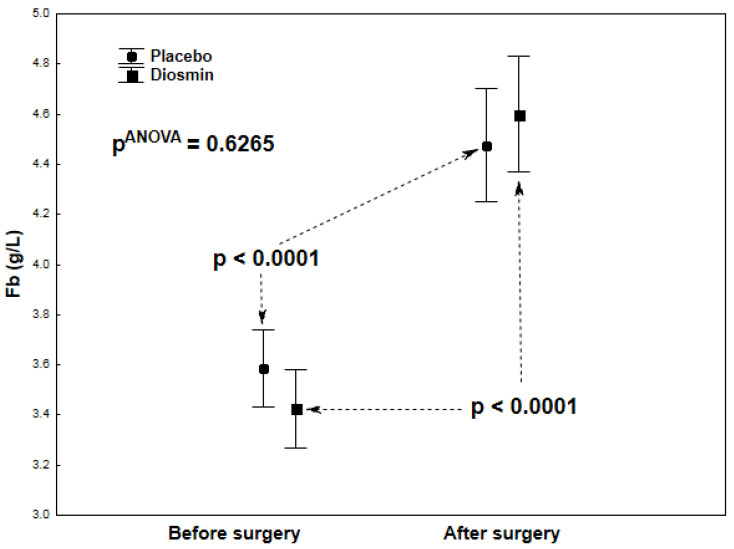
Fibrinogen (Fb) levels in patients who underwent acetylsalicylic acid therapy with diosmin supplementation (study group) versus patients who underwent acetylsalicylic acid therapy with a placebo (control group) at two times (before and after surgery).

There were no statistically significant differences in terms of demographic or clinical data between the study group (Diosmin) and the control group (Placebo) except for a significantly lower activity of the creatine kinase (CK-MB) isoenzyme in the study group (Table 1). Statistical analyses (each group tested in isolation) showed a significantly large decrease in platelet aggregation in the ASPI test (*p* < 0.0001) and a significant increase in fibrinogen concentrations (*p* < 0.0001) after surgery compared to the preoperative period, both in the study group (Table 2) and in the control group (Table 3). Similar changes were observed for CRP and IL-6. The study group (Table 2) showed a highly significant postoperative increase in both CRP (*p* < 0.0001) and IL-6 (*p* = 0.0092) concentrations. In the control group (Table 3), the postoperative increase in CRP concentration was also highly significant (*p* = 0.0003), while there was no significant increase in IL-6 concentrations (*p* = 0.1203). In both groups, there were no significant postoperative changes in platelet aggregation in the ADP test or platelet counts (PLT) (Table 2; Table 3). Two-way analyses of variance were also calculated to give *p*-values for interactions between group and assessment time, as shown in Figure 1A,B, Figure 2, Figure 3 and Figure 4.

As shown in Figure 1A, there was no interaction between group and assessment time (pANOVA = 0.9764) for arachidonic-acid-induced platelet aggregation (ASPI test). This indicates a similar ability in both groups to inhibit platelet aggregation in the ASPI tests, confirmed by the previously discussed results in Table 2 and Table 3. The ability of ASA to inhibit platelet aggregation in the ASPI test, regardless of group, was also confirmed by analysis of the frequency of platelet sensitivity to the anti-aggregating effect of ASA. It was shown that the percentage frequency of platelet sensitivity to ASA did not differ significantly between the groups, both before surgery (30% each) and after surgery (83% vs. 92%; *p* = 0.3292). With ADP-induced platelet aggregation (ADP test), no significant interaction was found between group and assessment time (Figure 1B; pANOVA = 0.1793), as shown by the results discussed in Table 2 and Table 3. However, in the post hoc tests (Figure 1B), the higher average platelet aggregation in the study (Diosmin) group compared to the control (Placebo) group before surgery (*p* = 0.059) is noteworthy, which, however, is not reflected by the sensitivity measurements. The percentage frequency of platelet sensitivity to ASA (ADP test) before surgery was non-significantly (*p* = 0.1731) lower in the study group compared to the control group (37% vs. 56%). Similarly, no intergroup difference was found in platelet sensitivity in the postoperative ADP test (50% vs. 42%; *p* = 0.5867). Analysis of variance by group type and assessment time also showed no interaction for platelet counts (pANOVA = 0.7612).

Figure 2, Figure 3 and Figure 4 show the results of the analysis of variance for the acute-phase markers (IL-6, CRP, Fb), the values of which are also given in Table 2 and Table 3. There were no significant interactions between groups (Diosmin/Placebo) and assessment times (before/after surgery) for IL-6 (pANOVA = 0.1487; Figure 2), CRP (pANOVA = 0.1611; Figure 3), and Fb (pANOVA = 0.6265; Figure 4). In general, it can be said that the postoperative increases in these acute-phase markers were not shown to be dependent on group. However, analyses of correlations between platelet function parameters and acute-phase markers showed significant relationships only in the study group and related only to the postoperative period: Highly significant positive correlations were found between platelet aggregation in the ASPI test and acute-phase proteins: with CRP (r = 0.85; *p* < 0.001) and with fibrinogen (r = 0.62; *p* = 0.001). Correlations were not significant for aggregation in the ADP test: with CRP (r = 0.38; *p* = 0.068) and with fibrinogen (r = 0.31; *p* = 0.135).

## 3. Discussion

As mentioned in the introduction, most patients who qualify for cardiac surgery using the CABG technique take antiplatelet drugs, most often ASA and less often clopidogrel. Antiplatelet therapy may result in imbalances in platelet hemostasis, of a hemorrhagic and/or a thrombotic nature. The dysfunction of platelet receptors responsible for platelet aggregation plays a key role in bleeding, as well as thrombocytopenia in extracorporeal circulation, during surgery [31]. Thrombotic events usually take the form of paroxysmal atrial fibrillation, one of the causes of which may be a low response to antiplatelet therapy [8,9,10]. Therefore, assessment of this treatment seems to be highly justified. As in the present study the patients did not show any hemorrhagic disorders due to thrombocytopenia (with normal platelet counts), the monitoring of antiplatelet therapy focused mainly on detecting sensitivity or resistance to ASA with or without diosmin supplementation. The best laboratory methods for monitoring antiplatelet therapy are to measure platelet aggregation by impedance aggregometry, with two laboratory tests recommended for this purpose: the ASPI test and the ADP test. The ASPI test is the recommended test for assessing the effectiveness of anti-aggregation treatment with acetylsalicylic acid, and the ADP test is recommended for the assessment of clopidogrel therapy [9,31,32]. As ADP is a physiological platelet agonist, its use in the present study was intended to give a baseline estimate of the degree of platelet activation in both groups of patients i.e., the diosmin supplementation group and the placebo control group, even though patients were not treated with clopidogrel.

Most flavonoids inhibit platelet aggregation through the same mechanisms as acetylsalicylic acid, by blocking the arachidonic acid pathway with consequent inhibition of platelet cyclooxygenase (=prostaglandin G/H synthase 1; COX-1) and thromboxane (TXA2) synthesis [16,17,33,34]. The results of the present study, however, do not confirm this assumed hypothesis about the potentiation of antiplatelet action of ASA by diosmin. This is indicated by the lack of significant differences between the levels of platelet aggregation and similar frequencies of platelet sensitivity in the group with diosmin supplementation versus the group that took the placebo. The fact that these observations concern both tests (ASPI and ADP) and both assessment times (pre- and postoperative) strengthens the conclusion that diosmin gave no additional antiplatelet effect. It should be emphasized that research into the anti-aggregation effect of flavonoids has sometimes been viewed as controversial due to differences in the bioavailability of flavonoids resulting from their origin (natural flavonoids contained in the diet or synthetic flavonoids) and also differences resulting from dose used in vitro or in vivo [14,15,17,32,34,35]. It should be emphasized that the anti-aggregation effect of flavonoids observed in vitro is associated with high doses that are impossible to achieve in vivo [15,32]. In our research, a potential reason for the lack of anti-aggregation properties of diosmin could be the use of too low a dose (1000 mg/day) and/or a short period of supplementation (30 days before to 30 days after surgery). The latest research on diosmin shows that supplementation at a dose only slightly higher than that used in the present study (2 × 600 mg/day), but taken for 3 months, inhibited the inflammatory reaction in patients with chronic venous disorders, as evidenced by a significant decrease in concentrations of IL-6 and tumor necrosis factor (TNF-alpha) [22]. In the present study, the lower activity of the CK-MB isoenzyme in the study group compared to controls (Table 1), indicating a lower degree of myocardial damage, may be indirect evidence of the anti-inflammatory effect of diosmin. Some studies have shown that the lack of a direct anti-aggregating effect of diosmin, as in our work, may be due to complex mechanisms of atherosclerosis pathogenesis. It is believed that flavonoids weaken the thrombotic-atherosclerotic and pro-inflammatory processes by modulating the aggregation function of platelets [33].

As mentioned earlier, the anti-inflammatory properties of flavonoids are well documented in both in vitro and in vivo studies, while in the case of anti-aggregation properties, literature reports diverge [15,17,32]. However, with regard to ASA, there are no doubts regarding anti-aggregation and anti-inflammatory properties and there are relatively well-defined relationships between them [25,36]. The anti-inflammatory effects of anti-platelet drugs are believed to be mediated by the inhibition of platelet function [36]. In the present study, indirect evidence of the relationship of platelet aggregation with inflammatory processes may be provided by the presence of a strong positive correlation between the size of platelet aggregation in the ASPI test and the concentrations of acute-phase markers, mainly C-reactive protein and fibrinogen, observed only in the group with diosmin in the postoperative assessment. This seems to be of interest: It is worth noting that, instead of the expected decrease in acute-phase proteins, there was a highly significant increase in the concentrations of all measured inflammatory markers, i.e., IL-6, CRP, and fibrinogen, compared to the assessment before surgery, in both groups of patients. This observation clearly indicates a lack of anti-inflammatory effect of diosmin as expressed by acute-phase proteins. This also shows that serum inflammatory markers are mainly characteristic of the postoperative acute phase and could be considered primarily as prognostic factors after CABG surgery. Many studies have shown that long-term inflammation after surgery is a risk factor for various complications, including thrombotic complications and mortality rate [25,37,38,39]. Interestingly, the degree of increase in CRP can be proportional to the scale of the postoperative inflammatory response [25]. In the present study, the results of platelet aggregation in the postoperative period deserve attention, perhaps resulting from the strong antiplatelet effect of ASA, especially in the preoperative period. Long-term postoperative inflammation may be a factor that promotes platelet activation via the mechanisms of increased thrombin generation [27], which in turn may potentiate the antiplatelet effects of ASA. Some researchers believe that the best anti-aggregation effects of ASA are achieved in the postoperative period [27,40]. When the strong links between inflammation and prothrombosis are considered, dual antiplatelet therapy with clopidogrel, for which anti-inflammatory effects have also been documented, should be considered to reduce the inflammatory response [41,42].

### Limitations of the Study

The limitations of the study are, firstly, the relatively small sample sizes and, secondly, the short-term period of supplementation with diosmin. This resulted from clinical procedures, and the presented results should be treated as an introduction to wider studies.

## 4. Materials and Methods

### 4.1. Subjects

The presented study was a prospective, randomized, double-blind study with two parallel groups. The study included 53 patients with coronary artery disease divided into two groups: a study group consisting of 26 patients taking flavonoids (diosmin at a dose of 1000 mg/day) and a control group consisting of 27 patients taking a placebo. Patient characteristics are presented in Table 1. The therapeutic period of diosmin ingestion started at least 30 days before and ended 30 days after coronary artery bypass surgery. Over the same period, all patients took acetylsalicylic acid (ASA) at a dose of 75 mg/day. Patients were recruited for the study based on inclusion/exclusion criteria during a visit to the Cardiac Surgery Clinic (Pomeranian Medical University, PUM, Szczecin, Poland), where they were qualified for coronary bypass surgery with the use of a saphenous vein. The main criteria for inclusion in the study were a qualification for coronary bypass surgery with the use of a saphenous vein and informed, written consent of the patient to participate in the study. Exclusion criteria were patients with symptomatic chronic venous disease (Hawaii stage > C1), hypersensitivity to diosmin or other flavonoids or hypersensitivity to ASA, alcohol addiction, renal failure (glomerular filtration rate (GFR) < 50 mL/min/1.73 m^2^) or liver failure (alanine transaminase (ALT) and/or aspartate transaminase (AST) activity > 3 times the upper reference range), inflammation (CRP > 5 mg/L), or a history of venous thrombosis treated with anticoagulants. The study was conducted according to the guidelines of the Declaration of Helsinki and approved by the Bioethics Committee at Pomeranian Medical University (KB-0012/04/16). Informed consent was obtained from all subjects involved in the study.

### 4.2. Blood Collection and Laboratory Analyses

Blood samples were taken from the vein of one elbow after fasting, into four different tubes before and 30 days after surgery. Tubes No. 1 contained 5 mL of blood with hirudin, for platelet aggregation determination in whole blood. Tubes No. 2 contained 3 mL of blood for serum, for the determination of IL-6 and CRP. Tubes No. 3 contained 5 mL of blood in 3.2% sodium citrate at a ratio of 9:1. These were centrifuged (15 min, 1500× *g*) to obtain platelet-poor plasma for the determination of fibrinogen (Fb) levels. Tubes no. 4 contained 2 mL of blood with EDTA to determine platelet counts (PLT). Platelet aggregation and PLT were assessed using fresh blood; the remaining parameters (IL-6, CRP, Fb) were determined from samples frozen at −30 °C.

### 4.3. Determination of Platelet Aggregation and Platelet Counts

Platelet aggregation was measured by impedance aggregometry (using Multiplate^®^ aggregometry, Dynabyte medical, Mannheim, Germany) less than 2 h after blood collection. Two types of assays were used for platelet aggregation measurements: the aspirin-induced platelet inhibition (ASPI) test (Roche Diagnostics GmbH, Mannheim, Germany), which used arachidonic acid as a platelet agonist (to monitor treatment response with ASA), and the ADP test (Roche Diagnostics GmbH, Mannheim, Germany), where adenosine 5′-diphosphate was the agonist (usually used to monitor treatment response with clopidogrel). The extent of platelet aggregation was expressed as the area under the curve (AUC) in arbitrary units (AU × min). To differentiate the platelet aggregation response, the cut-off points recommended by the manufacturer were adopted; for the ASPI assay, AUC > 300 indicated resistance and AUC ≤ 300 sensitivity; for the ADP assay, AUC > 500 indicated resistance and AUC ≤ 500 sensitivity. Platelet counts (PLT) were assessed (using an ABX Micros 60 hematology analyser; Horiba, Japan).

### 4.4. Determination of Interleukin-6, C-Reactive Protein, and Fibrinogen Levels

Serum concentrations of interleukin-6 and C-reactive protein were determined using an enzyme immunoassay method for IL-6 (IL-6 ELISA EIA-4640, DRG, Springfield, NJ, USA) and an immunoturbidimetric method for CRP (Cobas c 502 system, Roche/Hitachi, Basel, Switzerland). Plasma concentrations of fibrinogen were measured using a ready-made reagent kit (Q.F.A. Thrombin, ACL ELITE analyser, Instrumentation Laboratory Werfen, Barcelona, Spain).

### 4.5. Statistical Analyses

Data were statistically analyzed using commercial software (Statistica v.13.0; StatSoft, Tulsa, Ok, USA). Kołmogorov–Smirnov tests were used to test for normality and logarithmic transformations applied when necessary (for acute-phase markers). Unpaired Student’s *t*-tests were used to assess the effects of assessment time (pre- and postoperative) in the study (Diosmin) and control (Placebo) groups and a two-way ANOVA to assess interactions between the groups and treatment period (group × treatment time). Additionally, least significant difference tests were used post hoc. Chi-squared tests were used to estimate the prevalence of ASA sensitivity/resistance. Correlations were assessed between platelet parameters and acute-phase markers using Pearson’s correlation coefficients. A *p*-value of <0.05 was considered significant.

## 5. Conclusions

In conclusion, diosmin did not enhance the anti-aggregating effect of ASA in patients with coronary artery disease undergoing coronary bypass surgery, regardless of the time of assessment. A stronger ASA-induced anti-aggregation effect was found at the second assessment time, i.e., 30 days after surgery, independently of diosmin, and was associated with the acute phase of the postoperative period. The stronger anti-aggregating effect of acetylsalicylic acid in the postoperative period may be a response to prolonged inflammation, as indicated by persistently elevated levels of the acute-phase markers CRP, fibrinogen, and IL-6. Increased levels of inflammatory markers in the late phase of the postoperative period may provide an unfavorable prognostic factor in long-term follow-up, which should prompt the use of stronger antiplatelet therapy in patients after coronary bypass surgery.

## Figures and Tables

**Table 1:** Demographic and clinical data for groups of patients with coronary artery disease treated with cardiac surgery (coronary artery bypass grafts).

Values Either Mean ± SD or *N*/%	Diosmin *N* = *26*	Placebo *N* = *27*	*p*-Values
Age (years)	61.9 ± 6.4	64.7 ± 6.9	0.18
BMI (kg/m^2^)	29.4 ± 3.1	30.5 ± 4.8	0.63
GFR (mL/min/1.73 m^2^)	87.8 ± 13.9	84.2 ± 14.1	0.46
HGB (mmol/L)	8.7 ± 0.67	8.9 ± 0.56	0.26
CK-MB (U/L)	21.4 ± 7.7	26.0 ± 9.7	0.03
D-Dimers (ng/mL)	552 ± 472	707 ± 760	0.31
Gender, male	22/84.6	23/85.2	0.95
Hypertension	21/80.8	19/70.4	0.37
Diabetes	7/26.9	3/11.1	0.14
Hyperlipidemia	7/26.9	3/11.1	0.14
Cerebrovascular accidents	2/7.7	0/0.0	0.14
Atrial fibrillation	4/15.4	6/22.2	0.52
Lower extremity peripheral artery disease	1/3.8	2/7.4	0.57

Abbreviations: BMI, body mass index; CK-MB, isoenzyme MB of creatine kinase; GFR, glomerular filtration rate; HGB, hemoglobin; SD, standard deviation.

**Table 2:** Platelet aggregation and acute-phase markers in patients who underwent acetylsalicylic acid therapy with diosmin supplementation (study group) at two times (before and after surgery).

Parameter	Diosmin before Surgery (Mean ± SD)	Diosmin after Surgery (Mean ± SD)	*p*-Value
ASPI test (AU × min)	521 ± 255	236 ± 120	<0.0001
ADP test (AU × min)	644 ± 187	543 ± 368	0.1868
PLT (G/L)	240 ± 72	259 ± 82 *	0.3709
IL-6 (pg/mL)	30.7 ± 28.0	38.1 ± 25.4	0.0092
CRP (mg/L)	2.7 ± 3.8	15.1 ± 25.3	<0.0001
Fb (g/L)	3.43 ± 0.66	4.61 ± 1.49	<0.0001

Abbreviations: ASPI test, arachidonic-acid-induced platelet aggregation; AU, arbitrary units; ADP test, ADP-induced platelet aggregation; PLT, platelet count; IL-6, interleukin-6; CRP, C-reactive protein; Fb, fibrinogen; * 10 days after surgery; *p*-value, from paired Student’s *t*-test.

**Table 3:** Platelet aggregation and acute-phase markers from patients who underwent acetylsalicylic acid therapy without diosmin, i.e., with a placebo (control group) at two times (before and after surgery).

Parameter	Placebo before Surgery (Mean ± SD)	Placebo after Surgery (Mean ± SD)	*p*-Value
ASPI test (AU × min)	498 ± 263	210 ± 85	<0.0001
ADP test (AU × min)	486 ± 212	533 ± 355	0.6184
PLT (G/L)	219 ± 64	255 ± 126 *	0.1696
IL-6 (pg/mL)	38.6 ± 37.8	44.3 ± 36.2	0.1203
CRP (mg/L)	3.9 ± 5.2	8.4 ± 9.1	0.0003
Fb (g/L)	3.51 ± 0.83	4.44 ± 0.73	<0.0001

Abbreviations: ASPI test, arachidonic-acid-induced platelet aggregation; AU, arbitrary units; ADP test, ADP-induced platelet aggregation; PLT, platelet count; IL-6, interleukin-6; CRP, C-reactive protein; Fb, fibrinogen; * 10 days after surgery; *p*-value, from paired Student’s *t* test.

## Data Availability

Data are available on request due to restrictions, e.g., privacy or ethical. The data presented in this study are available on request from the corresponding author. The data are not publicly available due to reasons of sensitivity, e.g., human data.

## Sample Availability

Samples of the compounds are available from the authors.
